# Gamasid Ticks as Vectors of Tularemia in the Southeast of Armenia

**DOI:** 10.1089/vbz.2022.0082

**Published:** 2023-05-12

**Authors:** Arsen Manucharyan, Jenna Achenbach, Lusine Paronyan, Lilit Avetisyan, Ruben Danielyan, Gayane Melik-Andreasyan

**Affiliations:** ^1^ National Center for Disease Control and Prevention, Ministry of Health, Yerevan, Republic of Armenia.; ^2^ Battelle Memorial Institute, Charlottesville, Virginia, USA.

**Keywords:** gamasid ticks, tularemia, vectors, biodiversity, zoonotic disease

## Abstract

**Background:**

The natural environment of southeastern Armenia, which includes the Syunik and Vayots Dzor regions, provides a high biodiversity of flora and fauna, including ectoparasites. Currently, the fauna and ecology of gamasid ticks and their role in the circulation of tularemia in this area are unclear and incomplete. To better understand the persistence of tularemia in Armenia, an assessment of specific hosts and their vectors is needed to evaluate their role in perpetuating tularemia.

**Materials and Methods:**

Utilizing data and samples collected from 1970 to 2020, we have evaluated the species composition of gamasid ticks found on the common vole and in their nests and burrows, and identified the presence of tularemia over time. We evaluated five different geographical landscapes: semidesert, dry mountain steppe, mountain steppe, mountain forest, and high mountain in the communities and open areas of Kapan, Goris, Sisian, Meghri, and Jermuk.

**Results:**

We determined the density of gamasid ticks in southeastern Armenia over the 50-year period and isolated 20 cultures of tularemia in 12 separate years.

**Conclusions:**

It is important to regularly monitor gamasid ticks in southeastern Armenia to clarify the risk factors for the occurrence of tularemia epizootics, among both carriers and vectors, to better understand the full epidemiological picture.

## Introduction

The natural environment in southeastern Armenia provides a high biodiversity of flora and fauna, including many ectoparasites, various rodents, and small mammals. They are carriers, reservoirs, and vectors of various vector-borne diseases with a long history of tularemia.

The causative agent of tularemia, *Francisella tularensis,* is an especially dangerous zoonotic pathogen that can infect >300 species under natural conditions (Asanova et al., 2004; Friend, 2006; Olsufiev, 1975). The natural foci of tularemia are distributed across all continents in the northern hemisphere, including Europe, Asia, North America, and some countries of northern Africa along the Mediterranean coast. Tularemia is generally endemic between 30° and 71° north latitude (Beard and Dennis, 2010; Petersen et al., 2017).

Gamasid ticks are a poorly studied group of ectoparasites that are very diverse in biology and ecology. More than 98 species of gamasid ticks belonging to 10 families and 33 genera have been recorded in Armenia, and are both trophically and topically associated with small mammals (Hovhannesyan, 1966). Most of the species studied in Armenia are hematophagous nidikols living in the hairline of host animals, and in their burrows and nests (Evstafiev, 2017). Insufficient attention has been paid to the examination of gamasid ticks for the presence of *F. tularensis* as many researchers consider these parasites to be strictly mechanical vectors (Hopla, 1951; Zuevskiĭ, 1976).

The value of gamasids in natural ecosystems lies in their ability to support transmissible infections (Evstafév, 2015) by storing viable pathogens in their bodies for extended periods and in their potential to transfer from one vertebrate animal to another. Ticks of the *Hirstionyssus* genus, for example, *Haemolaelaps glasgowi* (Ewing), are found on a wide range of hosts, and are active members of the parasitic system that participate in the nest–burrow infectious transmission cycle. Therefore, they are more important in epizootological terms as carriers of tularemia from rodent to rodent participating in a small transmission cycle of disease (Nelzina and Barkov, 1951; Nelzina and Romanova, 1951; Nelzina et al., 1957).

The intensity of arthropod nest colonization depends on many factors, including the host species and its biological characteristics and the temporal and spatial (topical) factors of biotopic and zonal confinement. Generally, the nests of rodents are unevenly inhabited, and can contain anywhere from a few to thousands of gamasid ticks (Evstafév, 2015).

Among gamasid ticks, there are both oviparous (temporary parasites) and viviparous species. Unlike other groups of ticks, female gamasid ticks produce only one very large egg at a time which matures and is laid by the female within 3–8 h. Although gamasid ticks have low fertility, this is compensated for by the increased speed of development at all phases (Bregetova, 1956; Evstafév, 2015). In some parasitic ticks, the development of larvae or protonymph can occur inside the body of females within the eggshells. This contributes to a greater survival of ticks and accelerates their development.

In southeastern Armenia, the main carrier of tularemia is the common vole (*Microtus arvalis*), which is also the main host for gamasid ticks. This part of Armenia is dominated by two landscape zones: mountain steppe and Alpine high mountain.

During winter, the mountain-steppe zone of Armenia receives snow that partially covers the ground, and results in unfavorable living conditions for the common voles and their ectoparasites, causing both to decrease in numbers before the arrival of spring. In spring, gamasid ticks undergo intensive reproduction, and by the onset of summer the numbers continue to increase in the nests and on common voles reaching maximum levels by autumn.

By the end of autumn as cold weather returns, the reproduction of ticks on common voles and in their nests stops, and their numbers decline. During the common vole breeding season from March to October, secretions from the uterus combined with blood remain in the soil and litter of the nest providing food for ticks that feed on organic residues. The common vole breeds several times a year, which also coincides with tick activity and leads to an increase in the number of gamasid ticks throughout this period.

Alpine high-mountain zones are located at an altitude of 2200–3400 meters above sea level. Winter in this zone lasts from November through April and receives considerable snow. During this period, the common vole lives both in their main nests and in new nests built under the snow, which creates favorable conditions for the reproduction of gamasid ticks. Therefore, by spring the number of gamasid ticks on the common vole and in their nests is high. This is followed by a decrease in gamasid tick numbers through the summer.

The decrease of gamasid ticks during the summer period occurs after the snow melts and temperatures rise. The common voles arrange new nests carrying only those parasites on their bodies, and those ticks left in the abandoned nests eventually die. Once on the host and in the new nests, reproduction increases and numbers of gamasid ticks rise into autumn but never reach peak spring levels.

Thus, the highest number of gamasid ticks on the common vole and in their nests in the mountain-steppe zone is observed from the end of summer to the beginning of autumn and in the Alpine high-mountain zone in early spring. The species composition of gamasid ticks in the Alpine high-mountain zone is almost identical to the species in the mountain-steppe zone.

We detected several species of gamasid ticks, including *Laelaps muris*, *Laelaps hilaris*, *Haemogamasus nidi*, *Eulaelaps stabularis,* and *H. glasgowi*, which are known reservoirs and carriers of tularemia (Lopatina et al., 1998; Starikov et al., 2020; Starikov et al., 2016; Stepanova and Timoshkov, 1992). Through constant feeding, these ticks can maintain an uninterrupted epizootic among their carrier–host populations, which can spillover into the human population.

The aim of our work was to study the fauna and ecology of gamasid ticks in southeastern Armenia where limited or no research has been conducted to date. This was achieved by collection and analysis of gamasid ticks across the various geographical zones with a focus on the mountain-steppe and Alpine high-mountain zones. This study aims to elucidate where and when gamasid ticks and their hosts are involved in the circulation of tularemia in southeastern Armenia.

## Materials and Methods

### Study area

Between 1970 and 2020, specialists of the epizootological laboratory of especially dangerous infections performed field and laboratory research in the Syunik and Vayots Dzor regions located in southeastern Armenia. Armenia is divided into 10 separate regions, and further subdivided into areas and sectors. Epizootological and laboratory work was carried out by the Sisian and Jermuk area epidemiological teams and three zoological and parasitological teams of the Armenian Anti-Plague Station and its Kapan branch.

Communities and open areas of Kapan, Goris, Sisian, Meghri, and Jermuk were surveyed. These areas cover five geographical landscape zones: semidesert, dry mountain steppe, mountain steppe, mountain forest, and Alpine high mountain. We utilized the warmer temperatures of May through October to conduct the field examinations. A collection of archived data from the Anti-Plague Station was also accessed and analyzed for this research.

### Specimen collection

Gamasid ticks were collected from wild rodents, including the common vole, and their burrows by performing rodent combing and using Tullgren's thermal light selector method. The method of combing rodents is as follows: the rodent is caught by the tail, hair is combed in the opposite direction of the fur growth, which removes arthropods from the animal. Next, mechanical separation of small arthropods from the natural burrow material was performed using Tullgren thermal light collector. In brief, thermal light forces the arthropods to move due to their avoidance of bright light, heat, and dryness.

The Tullgren thermal light collector consists of three parts: a body, a lid (to which a light bulb is attached), and a funnel on which a wire mesh is placed. Avoiding bright light and heat, arthropods pass through the holes of the wire mesh, fall into the glass container placed under the funnel; a glass container is placed under the lower hole of the collector, a bowl filled with water is placed under the glass container to prevent ticks from climbing out of the glass container.

### Species identification and isolation of pathogen

Ticks from the same location are put to sleep by exposure to ether vapors for 1–2 min, after which they are placed in a Petri dish and examined under a microscope at 8–10 × magnification to determine the gender and species using typing keys (Bregetova, 1956).

Ticks are then placed into test tubes according to the species (50–100 gamasid ticks are placed in each test tube according to the protocol). The test tubes are numbered and recorded in the relevant protocol, which indicates the location of collection and the number of rodents and their burrows according to the species. Then, each type of rodent and the number of its nests are classified. This process is repeated for ticks collected from each location.

The test tubes were then sent to the laboratory for microbiological examination by bioassay using suspensions made from each pool of gamasid ticks. In brief, 3–5 mL of tick suspension solution is added into each test tube, and the ticks in solution are grinded to create a homogeneous suspension. From each suspension, 0.2–0.5 mL is injected subcutaneously into white mice, after which the mice are monitored for 7–10 days. In the event of the death of an experimental animal, samples of lymph nodes, lungs, liver, spleen, and blood are collected for bacterial isolation.

In brief, the tissue samples are ground in a 0.9% physiological saline solution to create a suspension, which is plated on McCoy and Chapin medium made of yolk and FT agar (WOAH, 2018), and incubated for 24 h at 37°C to allow for bacterial growth. This suspension of organ tissues is also injected into white mice for a second bioassay to obtain a pure culture of tularemia. Institutional and national guidelines for the care and use of laboratory animals were followed. All laboratory work and transportation of potentially infectious materials followed international guidelines (U.S. DHHS et al., 2020; WHO, 2020), and were approved by the National Center for Disease Control and the Health Ministry of Armenia.

### Spatial mapping analysis

A database was digitized and created from archival sources for the 50-year period. The database includes information on the types of carriers, vectors, and the number of detected tularemia cultures. Spatial and temporal data analysis and mapping were performed using ArcGIS version 10 (ESRI).

## Results

### Species collection and identification

During the period of 1970–2020, we collected 37,421 samples in the spring, 457,857 in summer, and 212,955 in autumn (Table 1). The gamasid ticks of southeastern Armenia were found both in the nests of and on the common vole in the mountain-steppe and Alpine high-mountain zones, and have the following species composition: *H. glasgowi*, *E. stabularis*, *L. hilaris*, *H. nidi*, *Macrocheles matrius*, *Macrocheles glaber*, *L. muris*, *Haemogamasus nidiformes*, *Laelaps agilis, Hyperlaelaps arvalis*, *Hirstionyssus criceti*, *Hypoaspis heselhausi*, *Hypoaspis praesternalis*, *Hypoaspis nolli*, *Androlaelaps fahrenholzi*, *Laelaps jettmari*, *Laelaps pitymydis*, *Haemogamasus bregetovae*, *Hirstionyssus isabellinus*, *Hirstionyssus latiscutatus*, *Hirstionyssus musculi*, *Hirstionyssus gudauricus*, and *Proctolaelaps pygmaeus.*

**Table 1.** The Number of Gamasid Ticks Collected by Year and Season with Pathogen Isolation

Year	Number of collected gamasid ticks			Number of isolated cultures by years	
	Spring	Summer	Autumn	Summer	Autumn
1970	11	1789	645	—	—
1971	504	3222	1429	—	—
1972	—	2675	30	—	—
1973	—	13,280	17,434	—	1
1974	—	15,203	12,853	—	—
1975	4473	37,869	16,012	—	—
1976	—	20,415	13,460	2	
1977	—	26,515	1723	1	
1978	—	16,547	20,381	—	—
1979	—	18,729	3304	—	—
1980	343	1609	3735	—	—
1981	—	5109	6000	—	—
1982	630	14,750	1309	—	—
1983	—	10,256	7124	2	1
1984	—	13,254	6243	—	—
1985	—	11,786	6183	—	—
1986	—	15,697	2045	2	
1987	—	9684	5660	—	—
1988	—	13,387	1665	—	—
1989	—	10,700	2245	—	—
1990	—	3505	800	—	—
1991	—	5840	2050	—	—
1992	—	1120	1553	—	—
1993	—	2586	815	—	—
1994	—	850	—	—	—
1995	428	235	—	—	—
1996	—	2410	1180	—	—
1997	—	2250	875	—	—
1998	—	325	687	—	—
1999	—	985	356	—	—
2000	—	4250	1420	—	—
2001	—	1170	540	—	—
2002	—	5208	—	—	—
2003	—	5775	2747	—	—
2004	—	4860	3050	1	
2005	—	4760	3980	—	—
2006	325	8127	3765	—	—
2007	350	8293	1422	—	—
2008	2310	7350	2200	—	—
2009	550	7550	5290	—	—
2010	1425	9825	3000	2	—
2011	3769	6350	5081	—	—
2012	773	3727	1779	—	—
2013	475	6690	2785	—	1
2014	—	11,450	4500	—	—
2015	1450	7970	4250	1	—
2016	1900	9900	9200	—	—
2017	7870	26,250	6800	—	2
2018	3685	11,770	5830	—	—
2019	4650	12,300	3300	—	2
2020	1500	11,700	4200	1	1
Total	37,121	457,857	212,935	12	8

### Pathogen identification

Over the 50-year period, tularemia was isolated from gamasid ticks in 12 separate years (Table 1). It should be noted that epizootological and laboratory studies in Armenia in the 1990s until the early 2000s were carried out in an incomplete manner due to lack of financing and war. Based on these difficulties, the data for these years do not fully reflect the complete epizootological picture of tularemia in Armenia.

In the 12 years with confirmed isolation of tularemia, 20 cultures were isolated from 92 pools of gamasid ticks (Table 2). Of the 20 tularemia isolates, 12 strains were obtained from the gamasid tick species *H. nidi*, 4 strains from *H. glasgowi*, 3 strains from *E. stabularis*, and 1 strain from *L. hilaris*.

**Table 2.** Results of Detection of Tularemia Culture in Pools with Gamasid Ticks by Geographical Location by Area and Sector

Year	Area, sector	Number of gamasid ticks by sectors positive for tularemia	Total number of pools	Positive pools	Number of gamasid ticks in one pool	Percentage of positive pools
1973	Sisian, Gorayk	1215	11	1	100	9
1976	Sisian, Gabur	876	7	2	150 100	28.5
1977	Sisian, Siskatar	1020	9	1	130	11.1
1983	Sisian, Spandaryan, Tsirnkar	1586	13	3	100 150 120	23
1986	Jermuk, Kechut	905	8	2	100 150	25
2004	Jermuk, Kechut	720	6	1	100	16.6
2010	Sisian, Berdalich	835	7	2	120 150	28.5
2013	Sisian, B. Karakhach	762	5	1	100	20
2015	Sisian, Akhlatyan	540	4	1	100	25
2017	Sisian, Agudi, Cguk	858	7	2	100 100	28.5
2019	Sisian, Tsirnkar, Tsguk	980	8	2	200 100	25
2020	Sisian, Tonasar, Siskatar	845	7	2	200 200	28.5

Based on >50 years of sampling, we found that our bacteriological cultures were more productive when the field material is examined <2 h after sampling and without freezing. We also noted that the years in which tularemia was isolated correlated with the highest numbers of recorded gamasid ticks.

Most tularemia cultures were isolated in the middle of summer and early autumn from gamasid ticks collected in sectors from the Sisian area in Syunik and in the adjacent sectors of the Jermuk area in Vayots Dzor. These areas share similar environmental conditions, and the time frame corresponds to the most favorable period for the reproduction and dispersal of the common vole, which is the main host of gamasid ticks.

**FIG. 1.**
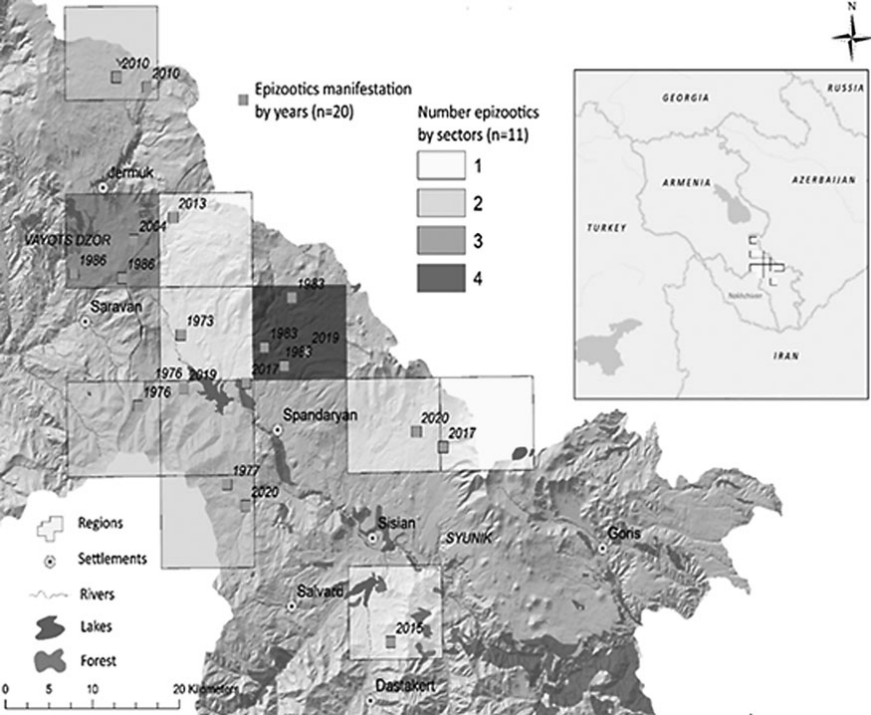
Identification of *Francisella tularensis* in gamasid ticks by sector, year, and numbers of epizootics.

Based on the spatial analysis of the distribution of gamasid ticks and isolated tularemia cultures identified by sector, we identified four cultures isolated in one sector in different years, three cultures in one sector, two cultures in four sectors, and one culture in five sectors since 1970 (Fig. 1).

## Discussion

The potential for gamasid ticks to be carriers of *F. tularensis* has attracted researchers' attention throughout the entire period of research on tularemia. Since many rodents are reservoirs of diseases, mites occurring on different host species may play an important role in epizootics and in the perpetuation of diseases, such as tularemia, rickettsial, and other infections (Baumstark et al., 2007; Bhuyan et al., 2016; Chaisiri et al., 2015; Di Palma et al., 2012; George et al., 2015). Several authors have underlined the role of acarians in the transmission of bacteria.

Among the species responsible for zoonoses, *F. tularensis* has been identified as being transmitted mechanically by various species of Dermanyssoidea such as Tabanidae, Simuliidae, or Ixodidae (Algazin and Bogdanov, 1978; Lysý et al., 1979; Petrov, 1971; Timofeeva, 1964; Zuevskiĭ, 1976). Some of these mites are clearly vectors for pathogens, but a great deal of study is still required to fully understand the vectorial role of these species.

From a biological point of view, we need to define the relationship between vectors and the pathogenic agents. This will require the development of models for gamasid ticks in the region using modern techniques of molecular biology for pathogen identification (Cortinas et al., 2002) and to study the host–pathogen life cycle in these ticks (Bruneau et al., 2001).

This is the first long-term assessment of the fauna and ecology of gamasid ticks as carriers of tularemia in southeastern Armenia. This area is one of the most active foci of plague and tularemia in the Transcaucasian high-mountain region with the development of tularemia epizootics during periods of absence of plague activity (Sludskij, 2014).

Our results and analysis show that of five areas in the southeast, the most stable in terms of tularemia activity is the Sisian area, which also produced the highest number of tularemia cultures isolated from gamasid ticks. Our long-term observations have shown that gamasid ticks are often infected with tularemia microbes. They are able to receive the pathogen through the alimentary route, preserve the pathogen internally, and potentially transmit tularemia in biotopes inhabited by the main carriers, although their ability to transmit tularemia requires further research.

The results show that the number of gamasid ticks in this region increases in the summer to autumn period, reaches its maximum by the third week of July, and remains stable until the first week of September. In addition, the isolation of tularemia cultures coincides with this period, after which a decrease in the numbers of gamasid ticks occurs.

We observed that the increase in gamasid ticks from July to September coincides with the peak of tularemia epizootics among common voles, and suggests that the role of gamasid ticks in maintaining tularemia epizootics may be significant.

To evaluate the possible correlation between the isolation of cultures from gamasid ticks on their number, we analyzed 670,792 gamasid ticks over 50 years. In the 12 years tularemia was isolated, a total of 234,929 (in autumn and summer) specimens were subjected to parasitological examination, which represents ∼35.02% of the total collection.

In Armenia, we have identified that the fauna of gamasid ticks in southeastern Armenia represents 40 different species of which we focused on 21 species that were specific to the common vole and their burrows.

We sought to compare our results from Syunik with studies in Iran as they share common borders, which revealed both similarities and differences in the composition of tick species. For example, Iran has detected five different species of the genus *Laelaps* (Gwiazdowicz et al., 2018), but have not reported *L. agilis* or *L. hilaris* which are both prevalent in Syunik and throughout Europe.

In addition, the parasitic mites of genus *Haemogamasus*, which are common in Syunik, Europe, and Northern Asia, have not been reported in Iran (Haitlinger, 1988). Conversly, *E. stabularis*, a parasitic mite that has been identified on >30 species of mammals and in nests of >30 species of birds, with a range covering Eurasia and North America (Bregetova, 1956; Masan and Fenda, 2010) was identified on unidentified birds in Iran (Gwiazdowicz et al., 2018) and on the common vole in Armenia.

The mountain-steppe zone, with its diversity of relief, flora, and fauna, is distinguished by the species diversity of gamasid ticks. Free-living forms and facultative blood-feeders are well represented by the families *Laelaptidae* and *Haemogamasidae*. The highest percentage of species in these two families are *H. nidi*, *H. glasgowi,* and *E. stabularis*. *L. hilaris* is the most prevalent wool parasite with *H. Criceti* being the most predominant obligate blood-feeder. For these species, the burrows of the common vole are a favorable place for both development and breeding.

The Alpine high-mountain zone is characterized by a reduced composition of gamasid tick species compared with the mountain-steppe zone. *H. nidi* was the most abundant species associated with the burrows of common voles, followed by *E. stabularis*, *H. glasgowi*, *H. criceti, and L. hilaris*. In total, five species were found both in burrows and on the common vole: *H. glasgowi*, *E. stabularis*, *L. hilaris*, *H. nidi,* and *H. criceti*, which represents 80.1% of the total collection.

The confinement of these ticks to the common vole and the isolation of tularemia cultures from them suggest that these species of gamasid ticks may be actively involved in the spread of *F. tularensis* and more research is necessary.

The majority of positive sectors are located in the same region, which confirms the high epidemiological potential of this territory. Indeed, over the period of 1996–2012, the Syunik region ranked second in the country in terms of the incidence of tularemia in humans (Melikjanyan et al., 2017). Additional analysis of epidemiological data from 2000 to 2012 showed that the Syunik region had the highest levels of tularemia compared with the rest of Armenia (Danielyan, 2014).

The role of these vectors in the epidemiology of tularemia and the continued survival and dissemination of tularemia remain to be defined. Continued research into these roles is vital due to the importance of zoonotic diseases to public health (Valiente Moro et al., 2005).

It is important to continue to monitor gamasid ticks in southeastern Armenia, and implement both vector and pathogen identification to fully understand the risk factors for the occurrence of tularemia epizootics among carriers and vectors in Armenia.
